# Immunoselected STRO-3^+^ mesenchymal precursor cells reduce inflammation and improve clinical outcomes in a large animal model of monoarthritis

**DOI:** 10.1186/s13287-016-0460-7

**Published:** 2017-02-07

**Authors:** Anwar Abdalmula, Laura M. Dooley, Claire Kaufman, Elizabeth A. Washington, Jacqueline V. House, Barbara A. Blacklaws, Peter Ghosh, Silviu Itescu, Simon R. Bailey, Wayne G. Kimpton

**Affiliations:** 1 Faculty of Veterinary and Agricultural Sciences, University of Melbourne, Parkville, VIC 5010 Australia; 2 Florey Institute of Neuroscience and Mental Health, Parkville, VIC 3010 Australia; 3 Department of Veterinary Medicine, University of Cambridge, Cambridge, CB3 0ES UK; 4 Mesoblast Ltd, 55 Collins Street, Melbourne, VIC 3000 Australia

**Keywords:** Animal model, Collagen-induced arthritis, Mesenchymal stem cells, Neutrophils

## Abstract

**Background:**

The purpose of this study was to investigate the therapeutic efficacy of intravenously administered immunoselected STRO-3 + mesenchymal precursor cells (MPCs) on clinical scores, joint pathology and cytokine production in an ovine model of monoarthritis.

**Methods:**

Monoarthritis was established in 16 adult merino sheep by administration of bovine type II collagen into the left hock joint following initial sensitization to this antigen. After 24 h, sheep were administered either 150 million allogeneic ovine MPCs (*n* = 8) or saline (*n* = 8) intravenously (IV). Lameness, joint swelling and pain were monitored and blood samples for leukocytes and cytokine levels were collected at intervals following arthritis induction. Animals were necropsied 14 days after arthritis induction and gross and histopathological evaluations were undertaken on tissues from the arthritic (left) and contralateral (right) joints.

**Results:**

MPC-treated sheep demonstrated significantly reduced clinical signs of lameness, joint pain and swelling compared with saline controls. They also showed decreased cartilage erosions, synovial stromal cell activation and angiogenesis. This was accompanied by decreased infiltration of the synovial tissues by CD4^+^ lymphocytes and CD14^+^ monocytes/macrophages. Over the 3 days following joint arthropathy induction, the numbers of neutrophils circulating in the blood and plasma concentrations of activin A were significantly reduced in animals administered MPCs.

**Conclusions:**

The results of this study have demonstrated the capacity of IV-administered MPCs to mitigate the clinical signs and some of the inflammatory mediators responsible for joint tissue destruction in a large animal model of monoarthritis.

## Background

Neutrophils play an important role in the initiation and progression of rheumatoid arthritis (RA) where they accumulate in large numbers within the synovium and synovial fluid (SF) of the joint affected joints [1–5]. Apart from their potent cytotoxic properties they also contribute to cytokine and chemokine release/activation and regulate immune responses through cell–cell interactions [3–5]. Recent research has also identified their ability to participate in autoimmune diseases via the production of neutrophil extracellular traps (Nets) [5]. Subsequent to the influx of neutrophils, there is an influx of monocytes that mature into tissue macrophages. Both cell types can play both pro- and anti-inflammatory roles [6]. In the autoimmune condition, rheumatoid arthritis, the prominent T cell infiltrate demonstrates that these cells are also key participants [7].

Murine models of arthritis have been widely used to identify many of the pathogenic pathways implicated in inflammation and joint tissue destruction in RA [4, 8–11], however, their high metabolic rate, low body mass and short lifecycle has resulted in some mechanistic discrepancies to human RA, particularly in their response to treatment with anti-arthritis therapeutic agents [11, 12].

The ovine model of collagen-induced arthritis (CIA) produces a reproducible model of inflammatory arthritis in hock joints over 2 weeks and this can be used to evaluate clinical signs of lameness, synovial fluid and synovial membrane changes and cartilage erosion through the course of the inflammatory response [13–15]. The major advantages of a large animal model such as the sheep is that the anatomy of the joints (in terms of tissue thickness) and the load-bearing stresses transmitted across the peripheral joints are comparable to the human. Since biomechanical factors are important contributors to joint tissue homeostasis and failure, we consider that assessment of lameness in this species is more relevant to the human disease than in rodents. Moreover, the ovine CIA model exhibits pain and swelling of the affected joints shortly after the induction of arthritis, followed by the development of a mild but chronic arthritis. From a practical standpoint, the ovine CIA model also has advantages over rodent models, with respect to the ease of access to target joints for intra-articular injections, collection of multiple fluid samples and the ability to cannulate the lymphatic ducts in order to monitor the cell populations exiting the joint [16].

Adult mesenchymal stem cells (MSCs) are able to differentiate into cells of the mesodermal lineage (including bone, cartilage and tendons) and were first developed therapeutically with a view to utilising their regenerative capacity [17]. However, a large number of studies have now reported the capacity of these cells to modulate immune system functions both in vivo and in vitro [18]. A limited number of studies of MSC therapy in rodent models of collagen-induced arthritis (CIA) have been published, reporting mixed therapeutic and non-therapeutic effects [19–24]. Differences in MSC source, isolation and culture techniques, dose, route and timing of administration may all account for the variable outcomes observed.

Nevertheless, several of these studies have reported promising therapeutic effects including suppression of T cell activation and reductions in pro-inflammatory cytokine production [19, 20, 22, 23]. Mesenchymal precursor cells (MPCs) are a restricted subset of MSCs that, when STRO-1 or STRO-3 immunoselected, demonstrate increased clonogenic, developmental and proliferative capacity compared with unfractionated MSCs [25].

In the present study, we utilized the ovine CIA model of monoarthritis to test the hypothesis that the intravenous administration of a single dose of 150 million allogeneic MPCs would reduce the clinical signs of arthritis, ameliorate the systemic elevation of leukocytes, particularly neutrophils, diminish the infiltration of inflammatory cells, synovial proliferation, stromal activation and cartilage destruction in the affected joints.

## Methods

### Animals

A total of 16 2-year-old female merino sheep were obtained from a local supplier and were acclimatised to their housing for at least 2 weeks before experiments commenced. The Animal Ethics Committee of the University of Melbourne approved all experimental animal procedures and sample collections (reference number: 1212422.3). One sheep from the MPC-treated group was excluded from the study at necropsy due to concurrent inflammatory disease of the lung that was unrelated to the study, giving final groups of eight control sheep and seven MPC-treated sheep.

### Arthritis induction

The ovine arthritis model was based on the sensitization of the animals to bovine collagen type II (BCII; [13, 26]). The sheep were allocated randomly into two groups. BCII was refined from bovine tracheal cartilage based on Miller’s method [27] using pepsin digestion (3200–4500 units/mg protein; Sigma-Aldrich, St. Louis, MO, USA) and fractional salt precipitation as we have described previously [13]. The purity of the preparation was confirmed by biochemical analysis for the hydroxyproline content and Western Blotting using a commercial sample of bovine tracheal cartilage type II collagen (Sigma-Aldrich). A single batch was then lyophilised and stored at -20 °C and used for all animals. Lyophilised BCII was dissolved aseptically in 50 mM acetic acid at 4 °C and reconstituted aliquots were stored at -80 °C. The dissolved collagen was emulsified with the Freund’s Complete Adjuvant (FCA) (Sigma-Aldrich) by mixing equal volumes using a Normject Luer-lock syringe (Henk Sass Wolf, Tuttlingen, Germany) connected to a second Normject Luer-lock syringe with a Popper micro-emulsifying needle. Sheep were sensitized to collagen on day 0 by subcutaneous injection in the flanks with an emulsion of 5 mg/ml BCII in Freund’s Complete Adjuvant (FCA). An immunization boost was given on day 14 by subcutaneous (SC) injection of 5 mg/ml BCII in Freund’s Incomplete Adjuvant (Sigma-Aldrich). Two weeks later (day 28), arthritis was induced by intra-articular (IA) injection of 100 μg BCII dissolved in 0.5 ml saline into the left hock (tibio-tarsal) joint. Both groups of sheep had arthritis induced in the left hock.

### Mesenchymal precursor cell treatment

Allogeneic, STRO-3^+^ ovine mesenchymal precursor cells (MPC) were provided by Mesoblast Ltd (Melbourne, VIC, Australia) cryopreserved in ampoules containing 30 million MPC/ml in 4 ml ProFreeze®/DMSO/αMEM. Ovine STRO-3^+^ MPC were immunoselected and expanded in culture to passage P5 as previously described [28, 29].

Following thawing, cell counting (using a Neubauer haemocytometer), and determination of viability (trypan blue exclusion method), 150 million MPC were injected into a sterile 0.9% saline drip bag (100 ml) immediately prior to administration into the sheep. The cells were then administered systemically over 30 minutes via a pre-placed jugular intravenous (IV) catheter. A filter was placed in the giving set to trap any cell clumps. Control sheep received the equivalent volume of saline. Treatments were administered 1 day following the intra-articular BCII injection and arthritis induction.

### Clinical lameness scoring

Clinical lameness was assessed using a semi-quantitative scoring system, as described previously [14]. A 6-point (0–5) scale was used for lameness, and 4-point (0–3) scales were used for joint swelling and pain elicited on flexion of the hock. The lameness assessment included the parameters of behaviour, standing posture and gait. The clinical signs for joint swelling were assessed as 0 (none detectable), 1 (barely detectable, but present), 2 (clearly discernible swelling on palpitation) and 3 (very marked joint swelling). Pain on flexion was assessed as 0 (none elicited), 1 (slight discomfort on strong flexion), 2 (clear discomfort with strong flexion) and 3 (severe discomfort even with slight flexion and sheep very reluctant to flex the joint). Lameness, joint swelling and pain on flexion were assessed weekly until the IA collagen injections (day 28) and then on days 29, 30, 31, 32, 34, 36, and 42 after the IA injection. All investigators who participated remained blinded to the treatment group allocation.

### Sample collection

Blood was collected from the jugular vein weekly until day 28, then daily for 3 days following arthritis induction, then every 2–3 days thereafter.

Sheep were killed 2 weeks after the induction of arthritis (on day 42), and post mortem examinations were performed. Synovial membranes (SM), cartilage from the articular surface of the talus bone, and synovial fluid were collected at necropsy from all animals. Gross findings were recorded for each tissue and SM were collected from the dorsal region of the left and right joints. Part of the SM was fixed in 10% buffered formalin and sent to Gribbles Veterinary Laboratories, Melbourne, VIC, Australia for routine processing and staining (haematoxylin and eosin) for light microscopy. The other half of the SM was placed in OCT compound (Tissue-Tek, Sakura Finetek, Torrance, CA, USA), snap frozen in liquid nitrogen and stored at -80 °C for immunohistology.

### Blood and synovial fluid cytology

Total leukocyte numbers in blood and SF were obtained using an automated cell counter (Coulter Particle Counter, Model Z1; Beckman Coulter, Indianapolis, IN, USA) while the differential cell count was determined on Giemsa-stained blood smears or SF cytospots. The differential cell count was performed counting a minimum of 200 cells under a light microscope. Results are presented as number of cells/ml of blood or SF.

### Cytokine assays

The concentrations of interleukin (IL)-17A and activin A were measured in plasma and synovial fluid by sandwich enzyme-linked immunosorbent assay (ELISA). The concentration of IL-17A in plasma and SF was estimated using Bovine IL-17A VetSet™ ELISA Development Kit (Kingfisher Biotech, Inc, Saint Paul, MN, USA) according to the manufacturer’s instructions. The concentration of activin A in plasma and SF was estimated using Quantikine ELISA ready kits (R&D Systems, Minneapolis, MN, USA) according to the manufacturer’s instructions. IL-10 was estimated using anti-bovine IL-10 (cc318, Bio-Rad Laboratories, Hercules, CA, USA) and biotinylated anti-bovine IL-10 (cc320, Bio-Rad Laboratories) followed by ExtrAvidin-HRP (Sigma-Aldrich). The standard was recombinant ovine IL-10, produced as described previously [30]. Interferon (IFN)-γ was estimated using anti-bovine IFN-γ mAb (cc330, Bio-Rad Laboratories) and biotinylated anti-bovine IFN-γ mAb (cc302, Bio-Rad Laboratories) followed by ExtrAvidin-HRP (Sigma-Aldrich). The standard was recombinant IFN-γ (23000-BG, R&D Systems). All assays were developed with TMB substrate (Life Technologies, Carlsbad, CA, USA). 

### Macroscopic scoring of articular cartilage from hock joints

The cartilage on the surface of the talus was assessed using a 5-point scale based on the Osteoarthritis Research Society International (OARSI) recommendations for macroscopic scoring of cartilage pathology [31], but simplified because the primary cartilage injuries were confined to the central trochlear groove of the talus, rather than the whole joint surface as we have described previously [14]. The scheme used was: normal cartilage surface = 0; roughened cartilage surface but not deep or extensive fissuring = 1; clear fibrillation and fissuring of surface = 2; full-depth small erosion confined to trochlear groove = 3; full-depth erosions which extended outside the trochlear groove to the adjacent condyles = 4. The mean of the values from two blinded independent observers were pooled for each treatment group.

### Histopathological scoring of synovial tissues from hock joints

The scoring system developed for this ovine CIA model was a composite of those used for humans with modifications for ruminants [31–34], and has been described previously [14]. The scoring system assessed three parameters of the synovium; namely intimal hyperplasia, stromal activation and inflammatory infiltrate (scores from 0 to 3) and the final score was a total of these three scores, with a maximal score of 9 points. For consistency, synovial intimal hyperplasia was specifically evaluated at the predominant cell depth [33]. The synovial intima ranged from normal (1–3 cells; 0 points) to moderate diffuse hyperplasia (>6 cells thick in multiple areas; 3 points). Synovial stromal activation and inflammatory infiltrate were based upon those areas with the greatest alterations [35]. The degree of activation ranged from none (0 points) to marked activation with chronic oedema, marked fibrosis and cellularity, including endothelial cells and histiocytes (3 points). Synovial inflammatory infiltration could vary from absence of inflammation (0 points) to marked inflammatory infiltrate, which could include lymphocytes, plasma cells and histiocytes, seen as large aggregations of cells within the synovial stroma, the synovial intima and in a perivascular location (3 points). Large areas of necrosis often accompanied severe inflammation. Each parameter was observed at low power, before evaluation at high power (×40 objectives). The pathological changes were scored by two blinded observers and if their scores differed by 2 or more points, the sections were examined by a third blinded observer.

### Immunohistochemical staining of synovial tissues from hock joints

Frozen sections of SM from the dorsal region of the arthritic and contralateral hock joints were acetone-fixed and stained by indirect immunohistochemistry. The primary monoclonal antibodies (mAbs) used were specific for CD4 (44-38), CD8 (38-65) [36] and γδ TCR (86D-127) [37] and were also obtained from A/Prof. Scheerlinck (Centre for Animal Biotechnology). B lymphocytes were identified using mAb specific for CD79acy (HM57, Dako, Glostrup, Denmark) and monocytes and macrophages were identified using mAb specific for CD14 (M-M9, VMRD, Pullman, WA, Australia). Monoclonal antibody specific for Ki-67 (MIB-1, Dako) was used to detect cells in active phases of the cell cycle while angiogenesis was detected using mAb specific for von Willebrand factor (vWF) (Dako) on endothelial cells. In all cases, isotype-matched non-specific antibodies were used as negative controls. The primary antibodies were detected with a rabbit anti-mouse HRP (Dako) and DAB (Sigma-Aldrich).

### Scoring of the immunohistochemically stained tissues from hock joints

The SM sections stained for mononuclear inflammatory cell types were scored and assessed on a 7-point scale (0–6) based on the approximate cell count and/or the size and the numbers of cell clusters [14]. The criteria used were: 0 = no cells in the entire section; 1 = < 10 cells in the entire section; 2 = 10–50 cells and/or 1–2 small clusters of cells; 3 = 50–200 cells and/or > 2 small–medium clusters of cells or numerous cells; 4 = 200–500 cells and/or many small–medium clusters; 5 = 500–1000 cells and/or many medium–large clusters of cells and 6 = > 1000 cells and/or very large clusters of cells. This system was modified for CD14+ cells, where the cell numbers were 10-fold greater (i.e. 0 to > 10,000 cells). The density of blood vessels in the synovium was estimated by counting the average number of vWF-stained vessels in three microscope fields using a × 4 objective.

### Statistical methods

Results are expressed as mean ± SEM unless otherwise indicated. Data were analysed using GraphPad Prism statistical software (version 6.0b; GraphPad Software Inc, La Jolla, CA, USA). Analysis of data between groups at different time points was performed using two-way ANOVA with Sidak’s multiple comparison tests. Area under the curve with respect to increase (AUC_I_) was used to compare changes in plasma cytokines where initial baseline values differed between groups. The AUC_I_ was calculated by the trapezoid rule using GraphPad Prism software, starting from day 29 (immediately prior to MPC or saline administration) and using the value at that point as the baseline. Endpoint data were evaluated using Mann-Whitney or Wilcoxon matched-pairs signed rank tests and statistical significance between groups was accepted at *p* < 0.05.

## Results

### Clinical assessment

**Fig. 1:**
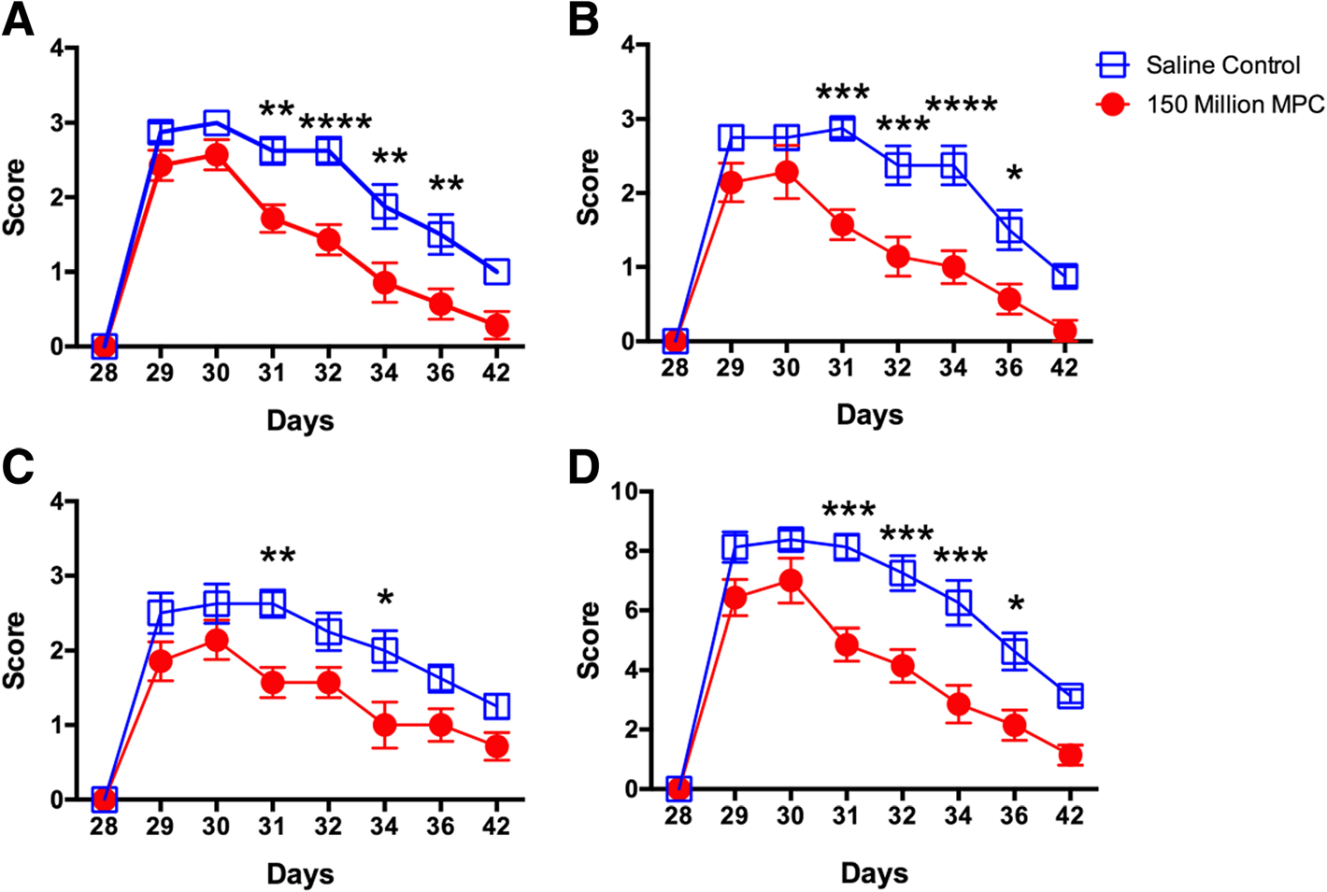
Effect of MPC administration on lameness (**A**), pain on joint flexion (**B**) and joint swelling (**C**) in sheep with CIA in the left hock. Intra-articular injection of BCII was performed on day 28 and MPC administration on day 29. There was a significant reduction in all measured parameters and the aggregate score (**D**) 2 days after MPC treatment. Values were analysed using two-way ANOVA with Sidak’s multiple comparison tests and each point represents the mean ± SEM of seven to eight sheep with ^*^, ^**^, ^***^and ^****^ representing *p* ≤ 0.05, 0.01, 0.001 and 0.0001 respectively. *MPC* mesenchymal precursor cells

Intra-articular administration of collagen caused a mild to moderate lameness with localised joint swelling and pain on flexion, which was detectable in all sheep after 24 h (Fig. 1). All signs of lameness and inflammation then decreased steadily from day 29 to day 42. Lameness scores were significantly lower in the group treated with MPC from days 31 to 36 inclusive (Fig. 1A), and there was a significant overall improvement in lameness for the MPC treatment group relative to saline controls. Lameness was decreased to a mean score of 1 by day 34 in the treated group while the untreated group still had a mean greater than 1 at day 42. For pain on flexion (Fig. 1B), there was a more rapid improvement in the MPC-treated group compared to the saline group with almost no pain by day 42. The pain scores were significantly improved between days 31 and 36 inclusive. Figure 1C shows the results of swelling in the hock joint. Although the swelling was mild, the scores for swelling were significantly lower for days 31 and 34 in the MPC-treated group. When combining all three clinical parameters (Fig. 1D), there were again significant improvements in the scores between days 31 and 36.

### Total and differential white cell counts (WCC) in blood

**Fig. 2:**
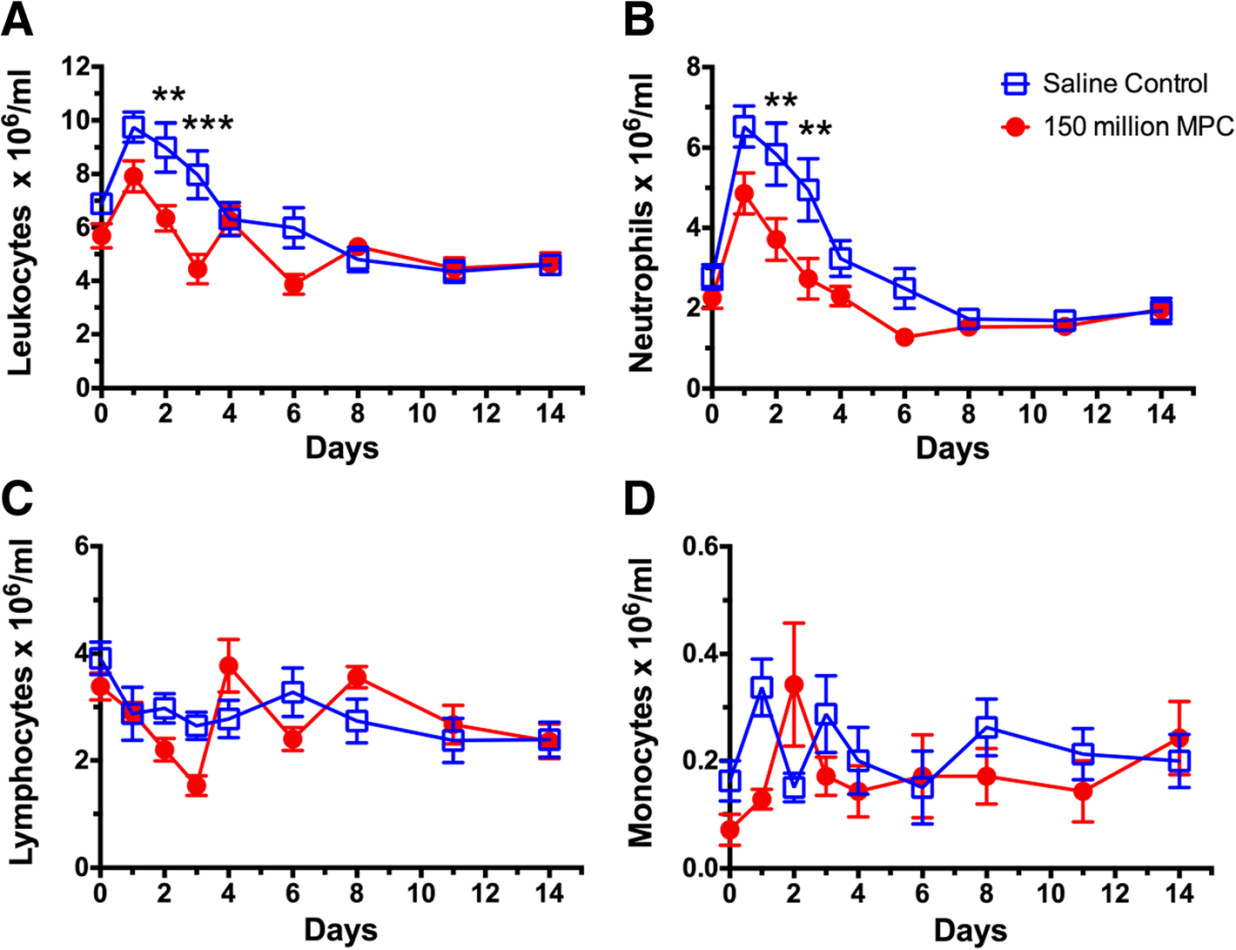
Effect of MPC administration on total blood leukocyte (**A**), neutrophil (**B**), lymphocyte (**C**) and monocyte (**D**) counts. Values were analysed using two-way ANOVA with Sidak’s multiple comparison tests and each point represents the mean ± SEM of seven to eight sheep with ^*^, ^**^ and ^***^ representing *p* ≤ 0.05, 0.01 and 0.001, respectively. *MPC* mesenchymal precursor cells

There was a significant increase in the total blood leukocyte count following IA arthritis induction in both treatment groups (Fig. 2A) and this was attributable primarily to the neutrophil numbers (Fig. 2B). However, following MPC treatment there was a faster decline in the number of neutrophils in the blood, with significantly lower neutrophil numbers measured for the 2 days following MPC infusion (Fig. 2A and B). Lymphocyte and monocyte numbers in blood showed little change after IA arthritis induction, and treatment had no effect on their numbers (data not shown).

### Total and differential white cell counts in synovial fluid

The total leukocyte count in the SF of arthritic left joints was significantly higher than in the contralateral right joints as we have previously reported [14], however the neutrophil counts were not significantly different between left and right joints in the MPC-treated sheep. After this period of time the leukocyte counts had decreased such that there were no significant differences between the synovial fluid cell numbers in the left hock joints of treated versus control sheep. However, there was a moderate trend to suggest that the numbers of neutrophils in the synovial fluid of the left hock joints in MPC-treated sheep (3 ± 1% of total cells) was reduced compared with the numbers in saline-treated control sheep (21 ± 8% of total cells; *p* = 0.09).

### Inflammatory markers in blood

#### Activin A

**Fig. 3:**
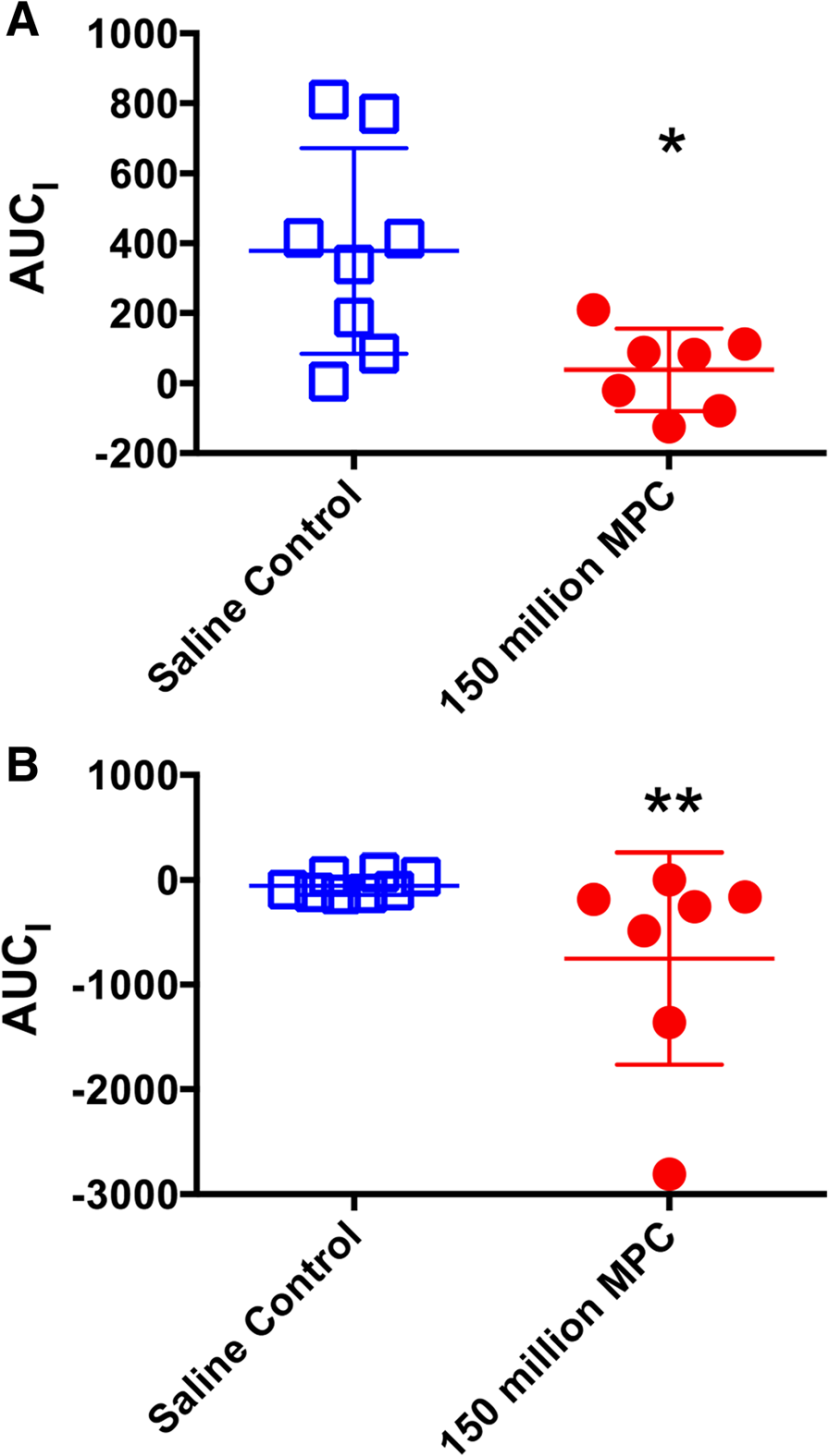
Effect of MPC administration on plasma cytokines in sheep with CIA. Plasma concentrations of activin A (**A**), and IL-17A (**B**) were compared between the MPC-treated and control group using two-way ANOVA with Sidak’s multiple comparison tests. The AUC_I_ for the maximal responses between days 29 and 36 of activin A (**A**) and IL-17A (**B**) were compared using Mann-Whitney tests. Each point represents the mean ± SEM for concentrations or mean ± SD for AUC of seven to eight sheep with ^*^and ^**^ representing *p* ≤ 0.05 and 0.01, respectively). *MPC* mesenchymal precursor cells

While the mean plasma activin A levels at individual time points for the two treatment groups were found not to be significantly different (due mainly to one sheep in the control group that had a 10–20-fold higher level of activin A than other sheep prior to arthritis induction) analysis of areas under the curve (AUC) for each sheep did demonstrate significant differences as shown in Fig. 3. Using this approach, starting from day 29 (immediately prior to MPC or saline administration), indicated that there was a significant drop in plasma activin A following MPC treatment (38 ± 117 arbitrary units; Fig. 3A) compared with plasma levels in untreated sheep (378 ± 294 arbitrary units; *p* = 0.021).

#### IL-17A

Plasma IL-17A levels decreased over the first 2 days post treatment in the sheep treated with MPCs. Again, there was variability between individual sheep in the initial baseline levels of this cytokine in both groups that precluded statistical significance using the mean values. However, analysing the area under the curve for IL-17A plasma levels for each sheep starting from day 29, demonstrated a significant reduction of this cytokine in the MPC treatment group compared to the saline-injected group (*p* = 0.006; Fig. 3B).

### Inflammatory markers in synovial fluid

**Fig. 4:**
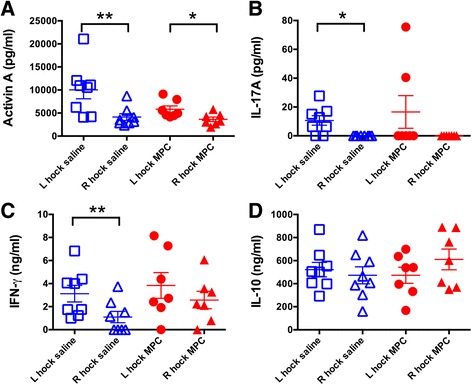
Effect of MPC administration on SF cytokines in sheep with CIA. Concentrations of activin A (**A**), IL-17A (**B**), IFN-γ (**C**) and IL-10 (**D**), were compared between the MPC-treated and the control group using paired Wilcoxon (left versus right) and unpaired Mann-Whitney (saline versus MPC treatment) tests. Lines represent mean ± SEM of seven to eight sheep and ^*^ and ^**^ represent *p* ≤ 0.05 and 0.01, respectively. *IFN* interferon, *IL* interleukin, *MPC* mesenchymal precursor cells

Although there were significantly raised levels of the cytokines activin A, IL-17A and IFN-γ, but not IL-10 in the synovial fluid of left arthritic joints compared to the right contralateral joints, no significant changes could be demonstrated between the saline and MPC treatments (Fig. 4A-C).

### Gross joint pathology

**Fig. 5:**
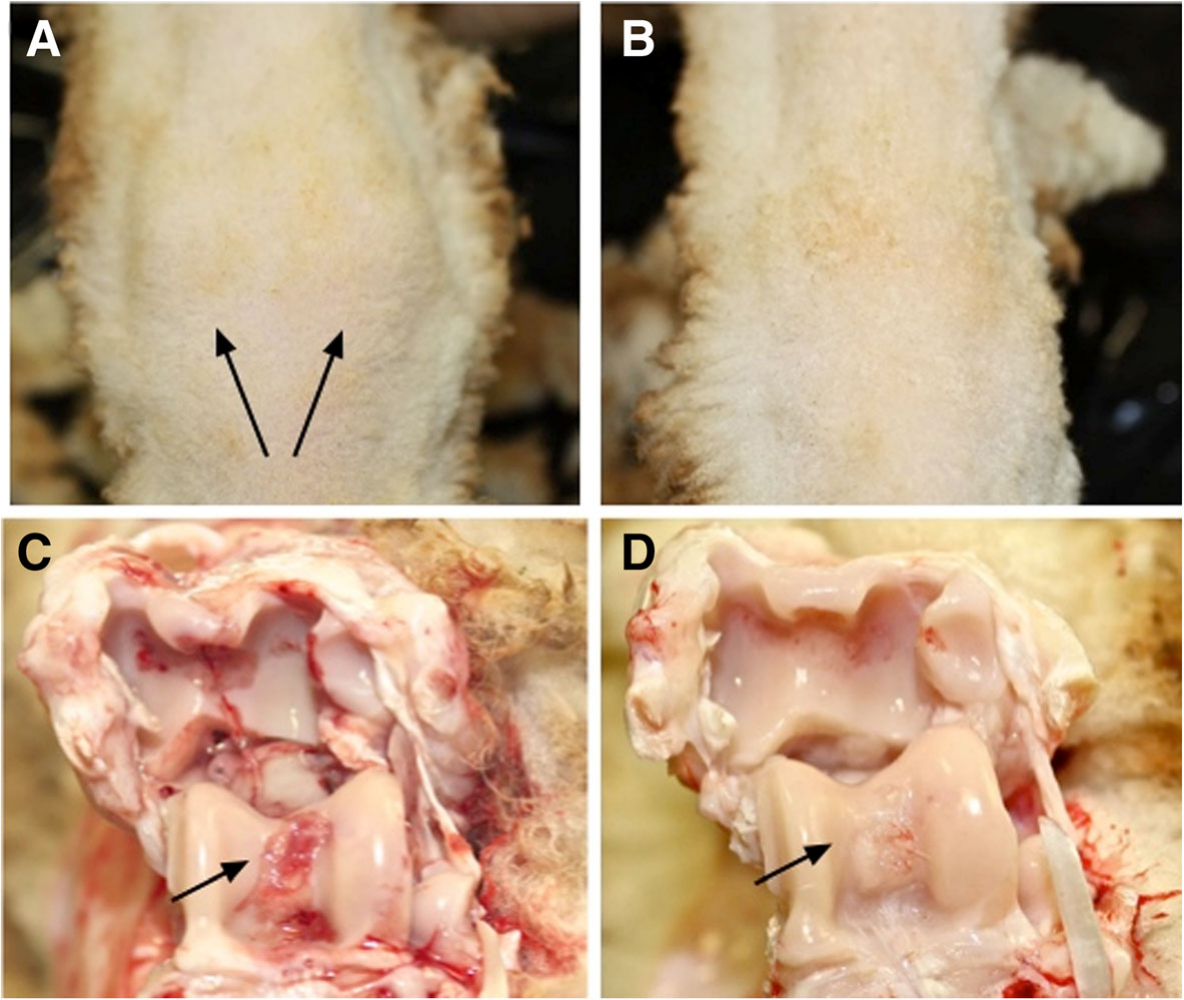
Effect of MPC treatment on gross joint pathology in the sheep CIA model. Representative photographs of joint swelling and gross appearance of articular cartilage from the left hock joints of control (**A** and **C**) and MPC-treated (**B** and **D**) sheep 14 days after the induction of arthritis, the arthritic left hock joint from the control animal shows more severe erosion of the cartilage on the articular surface of the talus bone (panel **C**; *arrow*) and on the articular surface of the distal tibia, compared to the MPC-treated sheep (panel **D**). *MPC* mesenchymal precursor cells

As shown in Fig. 5, at the time of necropsy (day 42), left hock joints revealed profound pathological changes. These included joint swelling, gross SM thickening and focal cartilage erosion on the articular surface of the talus bone and distal tibia bones. The magnitude of these changes were most evident in the left hock joints from the saline-treated control sheep (Fig. 5A and C) but were less pronounced in the MPC-injected sheep (Fig. 5B and D).

### Scoring of cartilage erosions

**Fig. 6:**
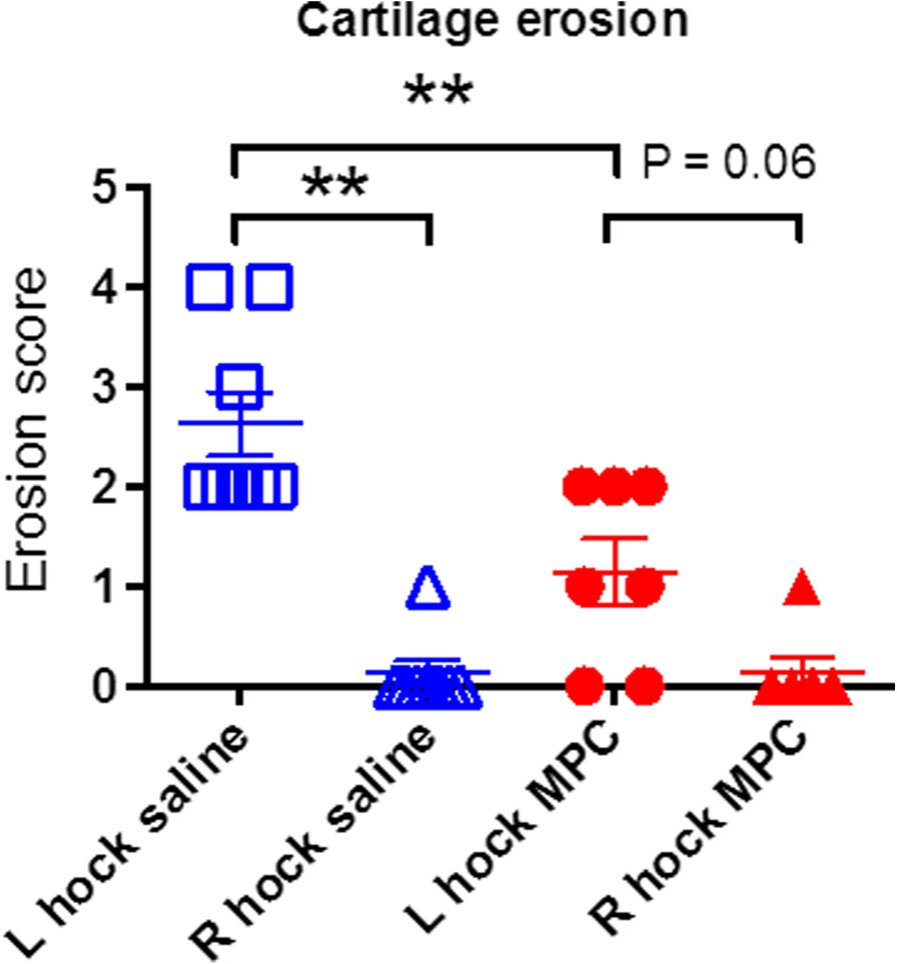
Effect of MPC treatment on cartilage erosion in the sheep CIA model. The cartilage on the surface of the talus was assessed macroscopically using a 5-point scale based on the OARSI recommendations for macroscopic scoring of cartilage pathology, as described in the Methods section. Values were compared between the MPC-treated and the control group using paired Wilcoxon (left and right) and unpaired Mann-Whitney (saline and MPC treatment) tests. Lines represent mean ± SEM of seven to eight sheep. ^**^ represents significant difference; *p* ≤ 0.01. *MPC* mesenchymal precursor cells

Cartilage erosions on the articular surface of the talus bone (Fig. 5A-D) were assessed using a macroscopic scoring system and MPC treatment was shown to significantly reduce cartilage erosion in the arthritic joint compared to arthritic joints from saline-treated control sheep (Fig. 6).

### Histopathological scoring of synovial tissues from hock joints

**Fig. 7:**
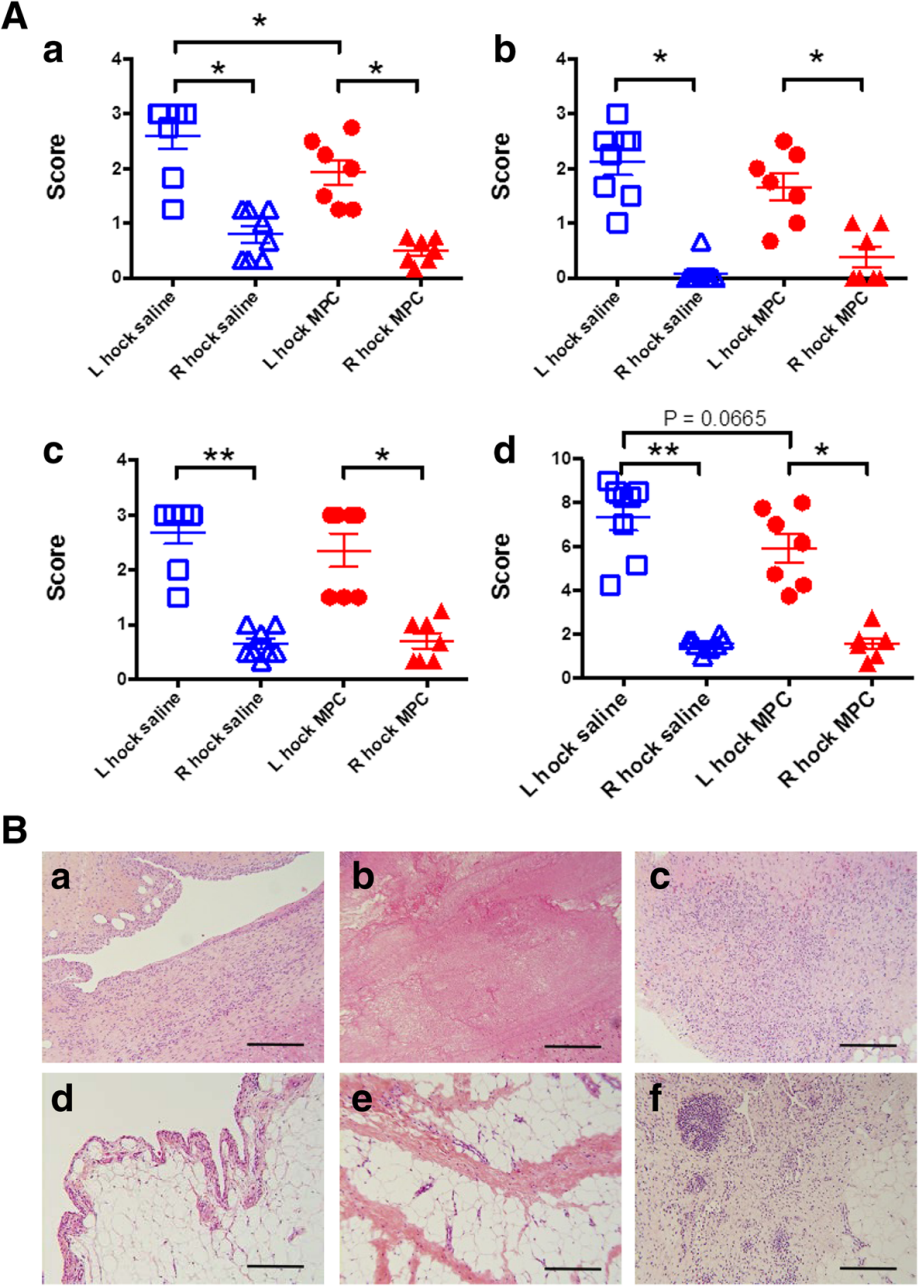
**A** Effect of MPC treatment on synovial histopathology in the sheep CIA model. Synovial histopathology scores were determined for samples from the left hock joints of control and MPC-treated sheep 14 days after the induction of arthritis. The parameters included stromal cell activation (*panel* ***a***), intimal hyperplasia (*panel* ***b***) and inflammatory cell infiltration (*panel* ***c***), plus a combined total score (*panel* ***d***). Values were compared between the MPC-treated and the control group using paired Wilcoxon (left and right) and unpaired Mann-Whitney (saline and MPC treatment) tests. Lines represent mean ± SEM of seven to eight sheep. ^*^ and ^**^ represent significant difference; *p* ≤ 0.05 and p ≤ 0.01, respectively. **B** Representative histopathological images of synovial membranes from arthritic hock joints following saline (***a*** *-* ***c***) or MPC (***d*** *-* ***f***) treatment. Paraffin-embedded tissues were stained with haematoxylin and eosin. The bars represent 250 μm. *MPC* mesenchymal precursor cells

The histopathology scores for synovial tissues, which included hyperplasia, stromal activation and inflammatory cell infiltration are shown in Fig. 7. All parameters differed significantly between the arthritic and contralateral synovial tissues within both groups. Treatment with MPC significantly reduced the levels of stromal activation, including lowered fibrosis, cellularity and matrix deposition, in the arthritic left joint compared to the same joint in the saline-treated controls (*p* < 0.05).

The reduction in intimal hyperplasia or inflammatory cell infiltration did not reach statistical significance. The combined total histopathology score also showed a trend towards being lower in the treated group (*p* = 0.0665; Fig. 7D).

### Immunohistochemical studies of the synovial tissues

**Fig. 8:**
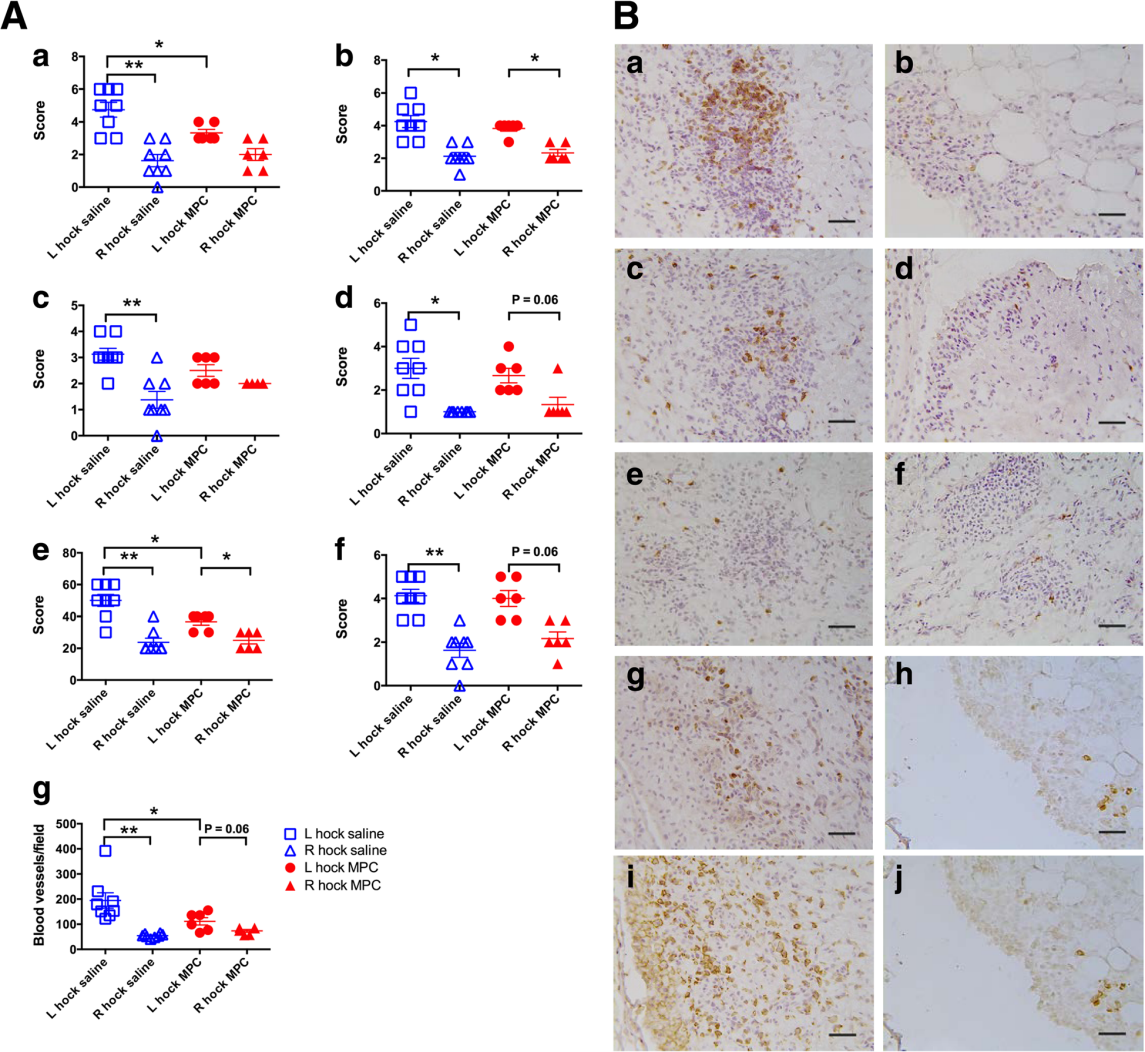
**A** Effect of MPC treatment on the number of inflammatory cells in SMs from sheep with CIA. Lines represent median and interquartile ranges of seven to eight sheep. Frozen sections were immunostained for CD4 (***a***), CD8 (***b***), γδ TCR (***c***), CD79a (***d***), CD14 (***e***) and Ki-67 (***f***). Blood vessels were identified by vWF expression (***g***). The data were compared using paired Wilcoxon (left and right) and unpaired Mann-Whitney (saline and MPC treatment) tests. Lines represent mean ± SEM of six to eight sheep with ^*^ and ^**^ representing *p* ≤ 0.05 and *p* ≤ 0.01, respectively. **B** Immunohistology of synovial membranes from arthritic hock joints following saline (*left column*) or MPC (*right column*) treatment. The frozen sections were stained with antibodies to CD4 (***a*** *,* ***b***), CD8 (***c*** *,* ***d***), γδTCR (***e*** *,* ***f***), CD79a for B cells (***g*** *,* ***h***) and CD14 (***i*** *,* ***j***). The bars represent 25 μm. *MPC* mesenchymal precursor cells

The inflammatory cell types (CD4^+^, CD8^+^ and γδ TCR^+^ T cells, B cells, and monocytes/macrophages) examined in the synovial tissues of the arthritic left joints were observed to be more abundant than in the corresponding tissues of the contralateral joints (Fig. 8a-e). Treatment with MPC significantly reduced the number of CD4^+^ T cells and CD14^+^ monocytes/macrophages (by 40%) in the synovium of MPC-treated sheep compared to the saline-treated control group (Fig. 8a, e). The other cell subsets (CD8^+^ and γδ TCR^+^ T cells, and CD79a^+^ B cells) were also lower in the MPC-injected group, but they were not statistically different from the saline-treated controls (Fig. 8b-e). Cells in active phases of the cell cycle, as indicated by Ki-67 expression, were also increased in the inflamed joints compared to contralateral joints, but showed no response to MPC treatment (Fig. 8f). There was, however, a significant reduction of 50% in the number of blood vessels (as measured by vWF expression) in the arthritic synovium of MPC-treated sheep compared with saline-treated controls (Fig. 8g).

## Discussion

This study evaluated the acute anti-inflammatory activities of immunoselected allogeneic STRO-3^+^ ovine mesenchymal precursor cells (MPCs) in an ovine model of monoarthritis and clearly demonstrated an anti-inflammatory effect of MPCs following IV administration. In rodent collagen arthritis models, where systemic administration of MSCs has reduced disease severity, this treatment has been associated with the suppression of T cell activation, a reduction in serum pro-inflammatory cytokine expression, and the induction of Foxp3^+^ regulatory T cells with an immunosuppressive phenotype [19, 22, 38]. MPCs are a restricted subset of MSCs, and this is the first study to demonstrate their effectiveness in a non-rodent, large animal model of collagen-induced arthritis.

The significant reduction in clinical scores within 4 days of systemic administration of the MPCs correlated with the marked decline of plasma neutrophil levels that were increased within 24 hours of arthritis induction. The increased blood neutrophil levels in response to CII immunisation have been attributed to the induction of IL-8 by IL-6, and the recruitment of neutrophils from the marginal pool by chemotactic factors including leukotriene B4, C5aR and FcγRs in sequence [4, 9, 10, 39]. However, in a parallel study using the same ovine model of CIA but sacrificing the animals 72 days post arthritis induction, leukocyte populations within synovial fluids were elevated, [14], but the levels were not altered by MPC treatment. Presumably, 2 weeks after arthritis induction, most of the inflammatory changes were restricted to the synovium itself, and there was little chemotactic stimulus to promote migration of neutrophils from synovium into the synovial fluid. Nevertheless, in the present study there was evidence to suggest that the numbers of neutrophils in the synovial fluid of the left hock joints in MPC-treated sheep was reduced compared with the numbers in joints of the saline-treated control sheep. Although SF samples were collected only at the time of necropsy, sampling at earlier time points in the study may have revealed differences in the synovial fluid cellular composition associated with MPC treatment; however this procedure was excluded from the study protocol due to the risk of inducing inflammatory changes within the joint from repeated joint sampling.

Activated neutrophils have been shown to be a major source of the inflammatory marker, activin A [40, 41]. Activin A is a member of the transforming growth factor beta (TGF-β) family, and it is thought to play a major role in inflammation. It stimulates the production of cytokines and iNOS, and regulates or suppresses TH1 and TH2 responses [42]. Increases in plasma concentrations of activin A have been demonstrated in patients suffering various inflammatory conditions [43]. In particular, activin A concentrations in the synovial fluid of patients with gout and rheumatoid arthritis are elevated relative to those patients with osteoarthritis [44]. In such conditions, it has been suggested that activin A promotes pro-inflammatory macrophages and induces hyperalgesia, and that early suppression of activin A in RA might reduce pain and joint damage [45]. In the present study, plasma activin A levels showed a significant increase in the saline-treated control group, soon after arthritis was initiated with a peak at day 34, while the sheep administered MPC exhibited relatively reduced levels of activin A. Our findings of lowered activin A levels, reduced blood neutrophils and lowered signs of pain on flexion and lameness in the MPC-treated group appears to support the hypothesis that the reduction of blood neutrophilia, with subsequent reduction of activin A and other pro-inflammatory factors, may be an important mechanism by which MPC exert their anti-inflammatory effect.

Plasma IL-17A levels decreased over the first 2 days post MPC treatment, however the IL-17A levels in blood were low and rather variable between individual sheep. IL-17A is a pro-inflammatory cytokine produced primarily by Th17 cells and γδ-T cells [46]. In gout and many other inflammatory conditions, assembly of intracellular pattern recognition receptors (NLRs) into the inflammasome complex leads to the enzymic activation of IL-1β and IL-18 into their mature forms, which promotes IL-17 production from the above mentioned cells [47]. In the joints of arthritic patients this cytokine is thought to promote inflammatory cell infiltration, bone destruction, and synovial fibroblastic activity [48–50]. Serum and synovial IL-17A levels in human RA patients can be significantly higher compared with normal controls, although IL-17A may not be present in all RA patients [51, 52].

MPC treatment did not appear to have a marked effect on cytokine levels in the synovial fluid of arthritic joints 2 weeks after arthritis induction. Activin A, IL-17A and IFN-γ were all higher in arthritic SF of the saline-treated sheep than in the contralateral joints, but not altered significantly in the MPC-treated group. Only IL-17A showed a possible reduction in SF of MPC-treated sheep, with no IL-17A detected in five sheep, but the effect of treatment was unclear due to the presence of two sheep that had very high levels of IL-17A.

The synovial membrane plays a very important role in disease pathogenesis in arthritis, with synovitis and erosion of articular cartilage being key features. The reduced cartilage damage evident in MPC-treated sheep is consistent with an anti-inflammatory mode of action. It is thought that cartilage damage is initially driven by inflammatory cytokines including IL-17A, IL-1β and tumour necrosis factor alpha (TNF-α), acting synergistically to induce the production of matrix metalloproteinase proteinases (including aggrecanases), which degrade cartilage [53, 54]. Reduced levels of stromal activation (fibroblast numbers, cellularity and matrix deposition) in the arthritic joints of MPC-treated sheep are important because the synovial stroma is the major site for inflammatory cell recruitment and cell activation.

Neutrophils play an important early role inflammatory arthritis, but in the current acute model they were no longer present in the synovium in significant numbers by the time of necropsy 14 days later, and instead lymphocytes, monocytes and macrophages were the predominant inflammatory cell types. MPC treatment 1 day after arthritis induction appeared to greatly limit the recruitment of CD4^+^ T cells and CD14^+^ monocytes and macrophages (by around 40%) to the synovium. CD4^+^ T cells and monocytes/macrophages both play an important role in the pathophysiology of arthritis. These cells release a number of chemotactic and other inflammatory cytokines such as TNF-α that recruit other inflammatory cells that maintain the arthritic process [55].

While MSC transplantation has been shown to suppress the proliferation of CD4^+^ T cells in a mouse model of graft-versus-host disease [56], it was uncertain whether the finding in the current study reflected reduced proliferation, or reduced recruitment of cells. Since the number of Ki-67^+^ cells was similar in saline- and MPC-treated synovium, it is arguable that reduced cellular recruitment may be a likely mechanism. This would be consistent with findings that MSCs significantly reduce expression of key chemokines for attracting macrophages (MCP-1) in vitro and in a rat model of acute traumatic brain injury, with a corresponding decrease in macrophage infiltration [57, 58].

Hyperplasia of the inflamed synovial tissue is supported by endothelial proliferation and angiogenesis, which in long-standing disease may ultimately result in the formation of an invasive pannus. Angiogenesis within the synovium was significantly reduced with MPC treatment in this study. Angiogenesis is a noted feature of RA and is thought to be associated with angiogenic chemokines and vascular endothelial growth factor [59–63]. VEGF is produced by monocytes, macrophages and fibroblasts; and together with IL-17A stimulates angiogenesis leading to cell recruitment and synovitis development [55, 64]. MSC are generally thought to increase angiogenesis via the release of VEGF [65] and naturally occurring MSC in the synovium of RA patients have been implicated in this process [66], so the significant reduction in angiogenesis following MPC treatment in the sheep model may be a specific effect of the highly purified MPCs used in this study.

## Conclusions

The results of the present study using the ovine model of inflammatory arthritis have confirmed that a single intravenous infusion of 150 million allogeneic MPC per animal was effective in reducing the clinical signs of arthritis and gross pathological changes in the arthritic joint. Treatment also attenuated histopathological changes including synovial stromal tissue activation, CD4^+^ T cell and monocyte/macrophage accumulation in the synovium and synovial angiogenesis. MPC treatment was associated with significant decreases in blood neutrophilia and associated inflammatory biomarkers such as activin A. This study demonstrates that MPCs were able to profoundly modulate the inflammatory cascade in this ovine model of collagen-induced arthritis, leading to a downregulation of both local and systemic inflammation. Together, these data support the potential application of MPCs for the treatment of acute tissue inflammation such as arthritis. It appears that MPCs have the ability to reduce the early events in the disease process, which is consistent with current recommendations for early anti-inflammatory intervention in arthritis [67].

The present studies also highlight the potential of using MPCs as a novel biological agent for the management of human RA. However, additional preclinical and clinical studies will be clearly required to establish more precisely the pathways used by MPCs to mediate their therapeutic effects in RA.

## Abbreviations

BCII: Bovine type II collagen
CIA: Collagen-induced arthritis
ELISA: Enzyme-linked immunosorbent assay
FCA: Freund’s complete adjuvant
IA: Intra-articular
IFN: Interferon
IL: Interleukin
IV: Intravenous
mAbs: Monoclonal antibodies
MCS: Mesenchymal stem cells
MPC: Mesenchymal precursor cells
RA: Rheumatoid arthritis
SF: Synovial fluid
SM: Synovial membrane
STRO-3: Stromal precursor antigen-3
TNF-α: Tumour necrosis factor
vWF: von Willebrand factor
